# Whole Genome Sequence of the Commercially Relevant Mushroom Strain *Agaricus bisporus* var. *bisporus* ARP23

**DOI:** 10.1534/g3.119.400563

**Published:** 2019-08-01

**Authors:** Eoin O’Connor, Jamie McGowan, Charley G. P. McCarthy, Aniça Amini, Helen Grogan, David A. Fitzpatrick

**Affiliations:** * Genome Evolution Laboratory, Department of Biology, Maynooth University, Maynooth, Co. Kildare, Ireland,; † Teagasc Food Research Centre, Ashtown, Dublin 15, D15 KN3K, Ireland,; ‡ Human Health Research Institute, Maynooth University, Maynooth, Co. Kildare, Ireland, and; § Sylvan-Somycel (ESSC - Unité 2), ZI SUD, rue Lavoisier, BP 25, 37130 Langeais, France

**Keywords:** Button mushroom, *Agaricus bisporus*, Genome report, Agaricus Resource Program, Agaricus *bisporus* mating locus, Agaricus pangenome

## Abstract

*Agaricus bisporus* is an extensively cultivated edible mushroom. Demand for cultivation is continuously growing and difficulties associated with breeding programs now means strains are effectively considered monoculture. While commercial growing practices are highly efficient and tightly controlled, the over-use of a single strain has led to a variety of disease outbreaks from a range of pathogens including bacteria, fungi and viruses. To address this, the Agaricus Resource Program (ARP) was set up to collect wild isolates from diverse geographical locations through a bounty-driven scheme to create a repository of wild *Agaricus* germplasm. One of the strains collected, *Agaricus bisporus* var. *bisporus* ARP23, has been crossed extensively with white commercial varieties leading to the generation of a novel hybrid with a dark brown pileus commonly referred to as ‘Heirloom’. Heirloom has been successfully implemented into commercial mushroom cultivation. In this study the whole genome of *Agaricus bisporus* var. *bisporus* ARP23 was sequenced and assembled with Illumina and PacBio sequencing technology. The final genome was found to be 33.49 Mb in length and have significant levels of synteny to other sequenced *Agaricus bisporus* strains. Overall, 13,030 putative protein coding genes were located and annotated. Relative to the other *A. bisporus* genomes that are currently available, *Agaricus bisporus* var. *bisporus* ARP23 is the largest *A. bisporus* strain in terms of gene number and genetic content sequenced to date. Comparative genomic analysis shows that the *A. bisporus* mating loci in unifactorial and unsurprisingly highly conserved between strains. The lignocellulolytic gene content of all *A. bisporus* strains compared is also very similar. Our results show that the pangenome structure of *A. bisporus* is quite diverse with between 60–70% of the total protein coding genes per strain considered as being orthologous and syntenically conserved. These analyses and the genome sequence described herein are the starting point for more detailed molecular analyses into the growth and phenotypical responses of *Agaricus bisporus* var. *bisporus* ARP23 when challenged with economically important mycoviruses.

The global market for edible mushrooms is estimated to be worth US$42 billion per year (Prescott *et al.* 2018). Due to its high yielding potential and appealing morphology the mushroom industry has relied solely on a single white variety of *A. bisporus*. However, the over-use of a single cultivar has led to a variety of disease outbreaks from a variety of pathogens (Hussey and Wyatt 1960; Fletcher 1992; North and Wuest 1993; Soler-Rivas 1999; Grogan *et al.* 2003). One approach to increase disease resistance profiles in important crops is the use of desirable traits from wild germplasms (Arora *et al.* 2019). However, breeding of new strains in the mushroom industry has been difficult. Notwithstanding these difficulties the introduction of desirable traits to novel cultivars and the need for genomic biorepositories of wild germplasms of closely related or ancestral agricultural crops remains crucial for the protection of low diversity crops (Dempewolf *et al.* 2017).

*A. bisporus* has effectively been considered a monoculture crop for a considerable amount of time (Royse and May 1982). To this end, the *Agaricus* Resource Program (ARP) (Callac *et al.* 1996) was set up to collect wild isolates from diverse geographical locations. The focus of this genome report is a wild isolate from the ARP collection referred to as *A. bisporus* var. *bisporus* ARP23. Crosses of homokaryons of U1 and old-fashioned brown led to an intermediate hybrid that was subsequently crossed with ARP23 to produce a novel commercially productive hybrid referred to as ‘Heirloom’. Wild strains are also a promising resource for the introduction of disease-resistant traits for common commercial mushroom diseases. It has been shown that the wild tetrasporic *A. bisporus* var. *burnettii* has heightened resistance to the pathogen that causes bacterial blotch (*Pseudomonas tolaasii*) through genetic markers linked to the *PPC1* allele (Moquet *et al.* 1999). Polygenic inheritance of resistance attributes has been described (Kerrigan 2000) and so the consideration of the introduction of wild *A. bisporus* germplasm into breeding novel strains must be considered on the basis of careful selection of screened wild strains with distinct mechanisms pertaining to disease resistance (Foulongne-Oriol *et al.* 2011).

To date, two genomes of the constituent homokaryons of Horst U1 have been sequenced, H97 (Morin *et al.* 2012) and H39 (Sonnenberg *et al.* 2016). This represents the genome of the first commercially cultivated white hybrid strain. The manuscript that reported the H97 genome sequence also described the genome of *Agarius bisporus* var. *burnetti* (JB137-S8), a strain exclusively native to the Sonoran Desert of California. That study uncovered the genetic and enzymatic mechanisms that favor *A. bisporus* to a humic-rich environment by primary degradation of plant material. A gene arsenal of compost-induced carbohydrate enzymes (heme-thiolate peroxidase, β-etherases, multicopper oxidase) and CYP450 oxidoreductases for example, together with high protein degradation and nitrogen-scavenging abilities were determined to be crucial to the challenges posed by complex composts (Morin *et al.* 2012).

The genome presented herein, represents the first commercially relevant genome for a wild cultivar of *Agaricus bisporus* var. *bisporus*, and is an invaluable tool for future efforts in mushroom breeding. Furthermore the genome sequence described will act as the starting point for more detailed OMIC based studies into the growth and phenotypical responses of *Agaricus bisporus* var. *bisporus* ARP23 when challenged with economically important mycoviruses.

## Methods

### Strain, culture conditions and homokaryon genotyping

*A. bisporus* var. *bisporus* ARP23 cultures were grown on compost extract agar (aqueous extract of phase II mushroom compost, double-autoclaved for sterility) for three weeks at 25° in the dark. 10 ml of protoplasting medium (50 mM maleic acid, 0.6 M saccharose, 1 M NaOH pH 5.8) containing 10 g Glucanex (Sigma Aldrich cat. no. L1412) (Kerrigan *et al.* 1994) was added to established mycelium and incubated for 5 hr at 25° in the dark with occasional shaking. Resulting protoplasts were grown on a saccharose-enriched compost extract medium and incubated for two weeks at 25° in the dark. To assess whether protoclones were homokaryons or heterokaryons, growth tests, hyphal morphology, and nuclear type were conducted. Cultures that had an average diameter of 54 mm (Kerrigan *et al.* 1994) or less after this time, were isolated as putative-homokaryons. A total of 86 out of 100 isolated protoplasts were assigned as putative homokaryons due to their delayed growth. Hyphae of homokaryons tend to be more ‘thread-like’ with far fewer branching hyphae. To confirm that protoplasts were homokaryons, the presence of a single MAT locus was assessed using the 39Tr 2/5-2/4 primer sets for targeting MAT loci and amplification was carried out as previously described (Gao *et al.* 2013). Genotype-validated homokaryons were grown on complete yeast media (2 g proteose peptone, 2 g yeast extract, 20 g glucose, 0.5 MgSO_4_, 0.46 g KH_2_PO_4_, 1 g K_2_HPO_4_, 10 g agar in 500 ml dH_2_0) for 3 weeks in the dark, at 25°. 7 mm agar plugs were excised from the growing hyphal edges of homokaryons of MAT2 genotype (the MAT1 genotype was not recovered) and shaken at 30 Hz for 7 min in 500 µL malt extract and the resulting homogenate added to 50 ml malt extract liquid medium (10 g malt extract in 600 ml of dH_2_O) in a 500 ml Erlenmeyer flask. Liquid cultures were grown for 12 days at 25° in the dark at 150 rpm.

### DNA isolation and libraries

Fungal mycelium was isolated in Miracloth and washed with sterile PBS. The mycelium was flash frozen and ground in liquid nitrogen using a mortar and pestle. DNA isolation was carried out immediately on ground material with the Wizard Genomic DNA Purification Kit (Promega) following the plant tissue method with minor modifications. Nuclei lysis buffer was supplemented with 0.5 M EDTA and 0.1 mg/mL Proteinase K and cell lysis was carried out at 37° for 30 min. The remainder of the DNA isolation was as per manufacturer’s guidelines. An Illumina paired-end sequencing library with insert size of 270 bp (80 X coverage) and a Pacbio RSII mate pair library of 20 Kb insert size were generated for a hybrid assembly approach. Sequencing on HiSeq 4000 and Pacbio RSII generated 5.90 GB and 57.64 GB of raw sequence data, respectively. DNA library construction and sequencing on the Pacbio (RSII) and Illumina (HiSeq 4000) platforms was carried out by BGI Tech Solutions Co., Ltd. (Hong Kong, China).

### Fruit-body material, RNA isolation and sequencing

ARP23 mycelia was added to mushroom compost in crates (*n* = 3) and incubated at 25°, 90–95% relative humidity (spawn-run phase) for 17 days. A layer of peat was added to the surfaces of the colonized compost (case-run phase) and incubated for another 7 days. Temperatures and relative humidity were lowered to 18° and 85–90% and fruit-bodies were harvested after 7 days of development. All cropping procedures were as per standard mushroom growing practices. Fruit-bodies were flash-frozen, freeze-dried and material was crushed in liquid N_2_. RNA was isolated using the RNeasy plant minikit (Qiagen) as per manufacturers guidelines. DNA digestion was done with DNase I (Invitrogen). RNA quantity and quality was assessed with an RNA6000 Nano Assay (Agilent 2100 Bioanalyzer, Agilent Technologies, USA). High-quality RNA was sent to BGI Tech Solutions Co., Ltd. (Hong Kong, China) for RNA sequencing (RNA-seq).

### Genome assembly and gene calling

Short-read libraries had adaptor removal (Martin 2011) and quality trimming performed using Trim Galore! (https://github.com/FelixKrueger/TrimGalore). A minimum phred score cut-off of 25 and a minimum read length of 90 nt was applied to short-read libraries. Long-read libraries were corrected using short reads using Proovread (Hackl *et al.* 2014). Corrected long reads were then self-corrected with additional adaptor trimming using Canu (Koren *et al.* 2017). Canu was then used for genome assembly with an error rate of 2.5%. Scaffolding of assembled contigs was performed by scaffolding with corrected long reads using SSPACE long-read hybrid assembler (Boetzer and Pirovano 2014). This primary assembly was then processed using Purge Haplotigs (Roach *et al.* 2018) for removal of duplicated haplotypes, with default parameters. These steps were carried out as a precaution to remove potential areas of heterozygosity in the assembly introduced by sequencing data collected from different MAT2 homokaron protoplasts, which were pooled for DNA isolation, due to their excessively slow growth rates in culture. Levels of heterozygosity were not explicitly examined however. Jellyfish v. 1.1.12 (Marçais and Kingsford 2011) was used to generate histograms of the frequency distribution of kmers from Illumina reads which were fed into GenomeScope (Vurture *et al.* 2017) for estimation of genome size. Synteny between the genome assemblies of ARP23, JB137-S8 and H97 was assessed by performing global whole genome alignments using BWA (Li and Durbin 2010) and visualized with Jupiter Plot (Chu 2018). The H39 assembly wasn’t included as it has been shown to have almost complete synteny to H97 (Sonnenberg *et al.* 2016).

For gene calling purposes, RNAseq data from fruitbodies with a read length of 100 nt (phred score > 25) were aligned to the final genome assembly using Bowtie 2 (Langmead and Salzberg 2012). Resulting alignment files were used to train AUGUSTUS using Braker2 (Stanke *et al.* 2006; Hoff *et al.* 2016). The completeness of the predicted gene models were assessed using BUSCO with the Basidiomycota BUSCO dataset (Simão *et al.* 2015).

### Genome functional annotation and characterization

Putative open reading frames (ORFs) were assigned protein family (PFam) domains using funannotate (https://github.com/nextgenusfs/funannotate) using default settings of Interproscan 5 (Jones *et al.* 2014). Gene ontology (GO) IDs (Ashburner *et al.* 2000) were assigned where available and a corresponding GO term map was obtained using YeastMine (Balakrishnan *et al.* 2012). Information on the pathways associated with different genes were analyzed with KEGG (Kyoto Encyclopedia of Genes and Genomes) (Ogata *et al.* 1999) by assigning KO (KEGG ontology) through BlastKOALA (Kanehisa *et al.* 2016). A search for repetitive elements was done by identifying tandem repeats (TR) and transposable elements (TE). TRs were identified over the entire assembly with Tandem Repeats Finder (TRF 4.07) (Benson 1999). Classification of the different categories of TE in the genome were conducted using RepeatMasker 4.06 with the modified version of NCBI Blast for RepeatMasker, RMBLAST (http://www.repeatmasker.org/RMBlast.html). Simple single repeats were also determined using RepeatMasker with the default settings. tRNAs were identified across all scaffolds using tRNAscan-SE v 2.0 (Lowe and Chan 2016) and rRNAs were also identified with RNAmmer 1.2 (Lagesen *et al.* 2007).

### Carbohydrate-active enzymes

Translated ORFs were used to search dbCAN2 (Zhang *et al.* 2018) for presence of Carbohydrate Active Enzymes (CAZys) (Lombard *et al.* 2014). For comparative purposes 32 fungal genomes consisting of a variety of Ascomycota and Basidiomycota species (Table S1) were also searched to catalog their CAZy content. As well as including brown rot and white rot fungi, genomes for 7 of the top 10 most highly cultivated mushrooms are also included in this dataset (Table S1).

### Mating locus

The locus coding for homeodomain proteins typical for the A mating type in *Coprinopsis cinerea* was used to locate the unifactorial *A. bisporus* mating-type locus. The homeodomain proteins as well as flanking proteins were individually searched using BLASTP (Altschul *et al.* 1997) (evalue 10^−3^) against the predicted proteomes of *A. bisporus* ARP23, H97 and JB137-S8 respectively. Top hits from these gene sets were then searched back against the *C. cinerea* gene set to locate reciprocal best BLAST hits which were then considered orthologs.

### Phylogenomic reconstruction

Orthologous gene families were identified with OrthoFinder2 (Emms and Kelly 2015), using BLASTp (Altschul *et al.* 1997) as the search algorithm, an inflation value of 2.0 for MCL clustering (Enright *et al.* 2002) and the command-line parameter “-msa”. 71 gene families were ubiquitously present and single copy and used for phylogenomic analysis. Each family was individually aligned using MUSCLE (Edgar 2004) and trimmed using trimAl (Capella-Gutierrez *et al.* 2009) with the parameter “-automated1” to remove poorly aligned regions. Trimmed alignments were concatenated together resulting in a final supermatrix alignment of 27,861 amino acids. Phylogenomic analyses were performed using both Maximum likelihood and Bayesian inference. IQ-TREE (Nguyen *et al.* 2015) was used to perform maximum likelihood analysis under the LG+F+R5 model, which was the best fit model according to ModelFinder (Kalyaanamoorthy *et al.* 2017), and 1,000 ultrafast bootstrap replicates (Hoang *et al.* 2018). Bayesian analyses were carried out using PhyloBayes with the CAT model (Lartillot *et al.* 2009). Two independent chains were run for 8,000 cycles and convergence was assessed using bpcomp and tracecomp. A consensus Bayesian phylogeny was generated with a burn-in of 10%. Support values represent posterior probabilities. The phylogeny was visualized and annotated using the Interactive Tree of Life (iTOL) (Letunic and Bork 2007).

### Agaricus bisporus pangenome dataset assembly

Genome assembly data for three *Agaricus bisporus* strains (H97, JB137 and H39) were obtained from NCBI. Gene sequence and genomic location datasets were generated for each of the three strains through the pangenome analysis pipeline Pangloss; a gene prediction strategy using a combination of HMM-dependent gene prediction with GeneMark-ES and PWM-dependent long ORF prediction was chosen (McCarthy and Fitzpatrick 2019b). Combined with data from *A. bisporus* ARP23, a total of 42,264 *A. bisporus* gene sequences and their corresponding genomic locations were predicted. An all-*vs.*-all BLASTp search was performed on the *A. bisporus* dataset using an e-value cutoff of 1e^-4^ (Camacho *et al.* 2009).

### Data availability

The Bioproject designation for this project is PRJNA544931. This Whole Genome Shotgun project has been deposited at DDBJ/ENA/GenBank under the accession VCNO00000000. The version described in this paper is version VCNO01000000.

Table S1 shows the genomes, taxonomy and download links for the 32 genomes used in the phylogenomic and CAZy studies. Table S2 shows the presence of lignocellulolytic genes in the 32 fungal genomes. Figure S1: Macrosynteny between *A. bisporus* H97 chromosomes and all *A. bisporus* ARP23 scaffolds. Only regions larger than 10,000bp are connected with links. Macrosynteny visualized with Jupiter Plot. Supplemental material available at FigShare: https://doi.org/10.25387/g3.9076991.

## Results

### Whole-genome assembly

**Figure 1:**
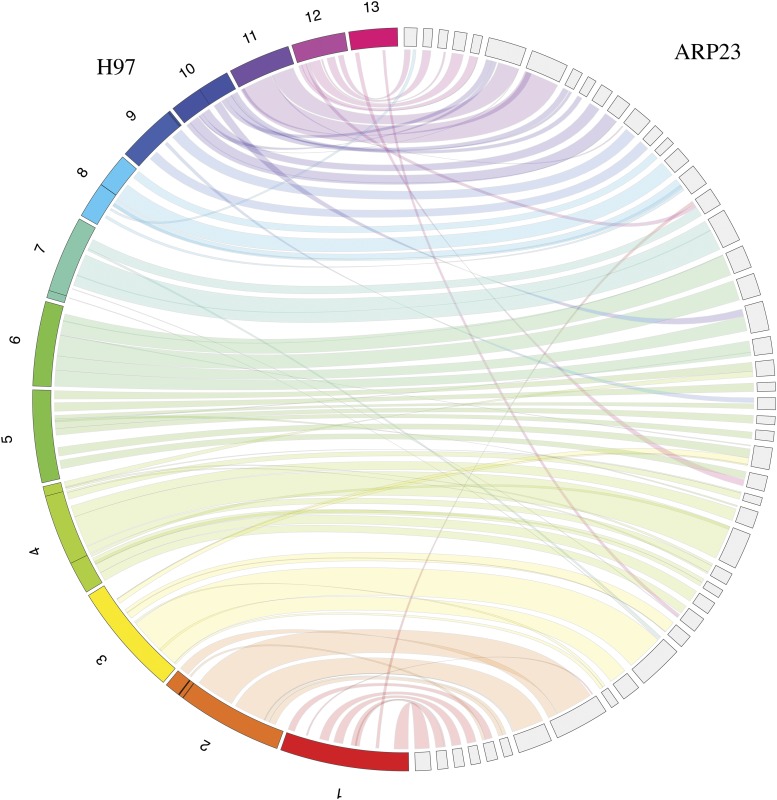
Macrosynteny between *A. bisporus* H97 chromosomes and *A. bisporus* ARP23 scaffolds. Only regions larger than 10,000bp are connected with links. Macrosynteny visualized with Jupiter Plot. For display purposes only the largest scaffolds that correspond to 75% of the ARP23 assembly are incorporated. When all scaffolds are included higher levels of coverage are observed particularly with respect to H97 chromosomes 1 and 13 (Figure S1).

The genome of the monokaryotic *A. bisporus* var. *bisporus* strain ARP23 (ARP23 herein) was sequenced using a hybrid approach of short (Illumina Hiseq 4000) paired-end reads and long-reads (Pacbio RSII). A total of 8,861,726 reads representing a cumulative size of 4.074 GB were generated including 8,424,105 and 437,621 reads from Illumina and PacBio sequencing platforms respectively. Upon trimming adaptors, error-correction and hybrid-assembly of both short and long-read libraries, a 33.49Mb genome with a GC content of 46.33% was generated. The assembly is comprised of 169 contigs, the longest being 1.5Mb with an N50 of 350,711 and an L50 of 26 (Table 1). The average length of contigs is 198,204.6 bp. Kmer-analyses conducted with GenomeScope (Vurture *et al.* 2017) suggest a genome size of 34.02Mb indicating that the 169 scaffolds of this assembly cover 98.44% of the entire genome. The completeness of the assembly was quantified by determining the presence/absence of the 1,315 fungal orthologs found in the Basidiomycete BUSCO set. A total of 1,170 (87.6%) complete BUSCO genes were located in the ARP23 assembly, this is comparable to what is observed in A. *bisporus* H97 (1,172 or 87.8%) and *A. bisporus* JB137-S8 (1,179 or 88.3%) strains (H97 and JB137-S8 respectively herin) (Table 1). Macrosynteny between the ARP23 assembly and the 13 complete H97 chromosomes was visualized with Jupiter Plot. Overall high levels of synteny are observed with the vast majority of ARP23 scaffolds mapping directly to individual H97 chromosome **(**Figure 1 & Figure S1**).** There are a number of scaffolds that have hits to multiple chromosomes indicating low levels of genome rearrangements have occurred (Figure 1 & Figure S1). There are no scaffolds in the ARP23 that do not map to the H97 assembly. Scaffold 74 was found to be 137,116 nucleotides in length and contains all 17 mitochondrial genes previously described in the mitochondrion of H97 (Férandon *et al.* 2013). Furthermore, it is also of a comparable length to the H97 mitochondrial genome (135,005 bp), therefore scaffold 74 corresponds to the full length ARP23 mitochondrial genome.

**Table 1:** Genome statistics for *A. bisporus* strains ARP23, H97 and JB137-S8

Feature	ARP23	H97	JB137-S8
**Number of Scaffolds**	169	29	2016
**Largest Contig**	1,506,893	3,343,696	2,973,556
**Total Size of Scaffolds (Mb)**	33.49	30.23	31.20
**N50**	350,711	2,334,609	1,225,131
**L50**	26	6	8
**GC content (%)**	46.33	46.48	46.59
**Number of Introns**	70,261	50,356	53,337
**Complete BUSCOs (C)***^a^*	87.6%	87.8%	88.3%
**Number of protein coding genes**	13,030	10,863	11,289
**Proteins with a signal peptide**	750	717	734
**Number of tRNA**	200	160	215
**Number of rRNA**	10	22	3
**Number of sRNA**	93	90	87
**Repetitive regions (%)**	0.79	0.79	0.75
**Number of tandem repeats**	2,287	2,353	2,480
**Simple repeat sequences (%)**	0.54	0.54	0.53
**Low complexity regions (%)**	0.13	0.13	0.12
**Non-LTR transposons**	250	240	134
**LTR transposons**	1	2	2

a BUSCO analysis conducted with the Basidiomycota (odb9) lineage.

### Genome annotation

The total length of repetitive elements in *A. bisporus* strain ARP23 amounted to 264,404 bp (0.79% of the total assembly) this is similar to what is observed for both H97 and JB137-S8 (Table 1). The number of tandem repeat regions across the 169 scaffolds of the assembled genome was 2,287, with ‘scaffold00003’ containing the greatest number at 321 repeat regions. Simple sequence repeats (SSRs) amounted to 180,995 bp (0.54%) and low complexity regions covered 44,108 bp (0.13%) (Table 1). We searched for the two sub-types of Non-LTR retrotransposons; long interspersed nuclear elements (LINEs) and short interspersed nuclear elements (SINEs). Of the 241 LINE-type elements; 17 belonged to L1, 69 to L2 and 78 to L3. For the 30 regions designated to SINES; 9 belonged to the MIRs and none were classified as ALUs. A single LTR retrotransposon was found for the endogenous retroviruses (ERV) class I. A single hAT-Charlie family DNA transposon and 7 DNA/TcMar-Tiggers were found out of a total of 55 DNA elements. Overall the ARP23 assembly contains 200 tRNAs, 10 rRNAs and 93 sRNAs (Table 1).

In total 13,030 putative protein-coding genes were called for ARP23. Of these 7,725 (59.3%) were annotated with PFam domains. An analysis of the presence of the BUSCO set of orthologous genes for Basidiomycetes in the ARP23 gene set revealed that is missing 40 (3%) of the BUSCO genes. This is comparable to the predicted proteomes of H97 and JB137-S8 which are both missing 39 (2.9%) of the BUSCO genes. ARP23 gene calling incorporated RNAseq data from fruitbodies, the final gene set showed evidence that 775 (5.95%) of protein coding genes are alternatively spliced. Overall the number of genes predicted for ARP23 is larger than that for H97 (10,863) and JB137-S8 (11,289) (Table 1). The average number of introns per ORF for the ARP gene set is 5.4, this number is larger than that observed in H97 (4.6) and JB137-S8 (4.7) respectively (Table 1). High-level functional annotations were assigned for predicted genes using BlastKOALA (Kanehisa *et al.* 2016). Of the 13,030 predicted ARP23 genes, KO assignments were made for 3,808 (29.22%). General functions and protein families relating to genetic information processing accounted for the majority of KEGG annotation with 1,693 (45.46%) of proteins falling into these categories. Other pathways highly represented were carbohydrate metabolism with 313 (8%) and environmental information processing with 176 (5%). A total of 750 secreted proteins were predicted by assigning signal peptides in SignalP v. 5. Putatively secreted proteins with a transmembrane domain downstream of the N-terminus signal peptide were excluded (Sonnhammer *et al.* 1998), with the subsequent prediction of 606 proteins. Secreted proteins involved in hydrolysis of glycoside (*n* = 54, GO:0004553), oxidation-reduction processes (*n* = 82, GO:0055114), and fungal hydrophobins (*n* = 20, PF01185) were highly represented.

### Genome phylogeny

**Figure 2:**
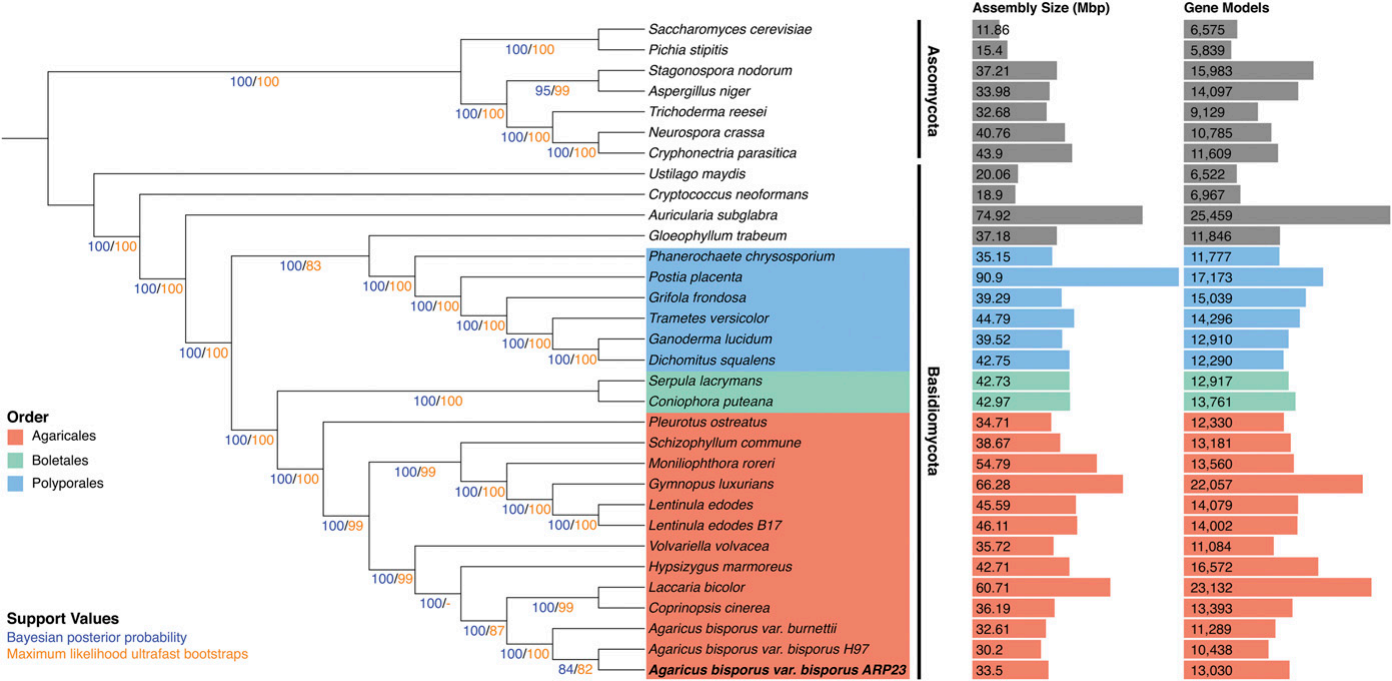
Supermatrix phylogeny of 32 fungal species (71 ubiquitous fungal gene families, 27,861 characters). Phylogenomic analyses were performed using both maximum likelihood (IQ-TREE with LG+F+R5 model) and Bayesian inference (PhyloBayes with the CAT model). Both phylogenies were identical except the maximum likelihood phylogeny grouped *V. volvacea* and *H. marmoreus* as sister taxa while the Bayesian phylogeny did not. Bayesian posterior probabilities and ultrafast bootstrap supports are indicated at all nodes. For comparative purposes the assembly size and number of gene models for each species are also shown.

The availability of whole genomes permits the reconstruction of phylogenomic trees. From our dataset of 32 fungal genomes we located 71 ubiquitously distributed gene families. These were individually aligned and concatenated to give a supermatrix of 27,861 amino acids. Using this supermatrix, phylogenomic reconstruction analyses were performed using both Maximum likelihood and Bayesian inference (Figure 2). The resultant phylogeny successfully resolved strongly supported monophyletic clades for the Ascomycota and Basidiomycota phyla. It also resolved monophyletic clades for the Agaricales, Boletales and Polyporales orders within the Basidiomycota clade (Figure 2). Within in the Agaricales order a strongly supported monophyletic Marasmioid clade containing *Schizophyllum commune*, *Moniliophthora roreri*, *Gymnopus luxurians* and *Lentinula* species is present. A monophyletic Agaricoid clade containing *Laccaria bicolor*, *Coprinopsis cinerea* and *Agaricus bisporus* is also present (Figure 2). The single Tricholomatoid clade species, *Hypsizygus marmoreus* is grouped beside the Agaricoid clade in agreement with previous studies (Matheny *et al.* 2006). However the two Pluteoid clade species, *Pleurotus ostreatus* and *Volvariella volvacea* are not grouped together, however it has been suggested that the Pluteoid clade may not be monophyletic (Matheny *et al.* 2006).

With respect to the phylogenetic relationships between the *A. bisporus* strains, our phylogeny groups all three strains in a monophyletic clade with maximum Bayesian posterior probability (BPP) and bootstrap support (BP). Furthermore, H97 and ARP23 are grouped as sister taxa with relatively strong BPP and BP (Figure 2). This phylogenetic relationship infers that ARP23 is more closely related to H97 that it is to JB137-S8.

### Carbohydrate-Active Enzymes (CAZys)

*A. bisporus* is adapted to growth in a humic-rich, leaf-litter environment. The genome of H97 has a carbohydrate-active enzyme gene (CAZyme) (Lombard *et al.* 2014) repertoire more similar to that of white- and brown-rot basidiomycetes as opposed to closer taxonomically-related species such as *C. cinerea* and *L. bicolor* (Figure 2) (Morin *et al.* 2012). Similar results were replicated in our analyses of the genomes of *A. bisporus* H97, JBS137-S8 and ARP23 (Table S2). A total of 411 putative CAZymes were found in the genome of ARP23 including 176 glycoside hydrolases, 60 glycosyl transferases and 14 carbohydrate-binding modules. In terms of lignocellulolytic genes the three strains of *A. bisporus* have very similar repertoires with ARP23, JB137-S8 and H39 having 149, 142 and 136 genes respectively. Specifically, ARP23 was found to contain 48 cellulases, 19 hemicellulases, 12 pectinases, 17 lignin oxidases and 53 lignocellulolytic auxiliary enzymes (Table S2).

### Mating locus

*Agaricus bisporus* has a pseudo-homothallic life cycle with a unifactorial mating system (Miller 1971; Raper *et al.* 1972). Pseudo-homothallism is a particular system in which automixis is forced, as two haploid nuclei from one meiotic tetrad are packaged together into one spore, therefore self-fertility is the result of the packaging of two independent and opposite mating type nuclei within a single spore (Wilson *et al.* 2015). This lifestyle not only enables the fungus to reproduce without finding a compatible partner, but also to cross with any compatible mate it may encounter (Grognet and Silar 2015). To date this type of reproduction in Basidiomycetes has only been observed in Agaricomycetes (Nieuwenhuis *et al.* 2013). It is possible that pseudo-homothallics benefit from both homothalism, which allows the possibility to self-cross when no compatible partner is present and heterothallism, which favors the creation of genetic variation through recombination during outbreeding (Grognet and Silar 2015).

**Figure 3:**
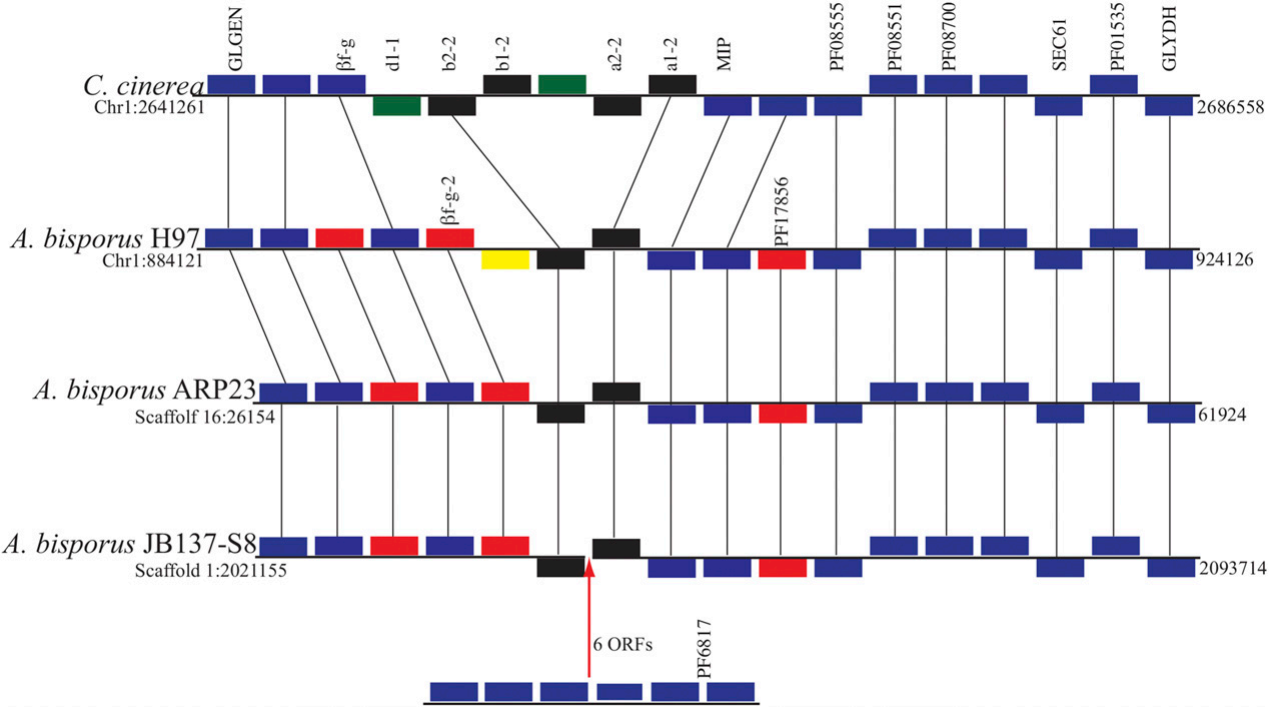
Distribution of genes in the mating type locus of *A. bisporus* H97, ARP23 and JB137-S8. For comparative purposes the mat A locus of the model species C. *cinerea* is also displayed. Chromosome or scaffold and relative genomic position of loci are indicated. Genes that are colored blue are orthologous in all four genomes and syntenically conserved. Red genes indicate syntenically conserved orthologs in *A. bisporus* strains but missing from *C. cinerea*. *A. bisporus* has a single pair of homeodomain genes (colored black) while *C. cinerea* has two pairs. Green genes in *C. cinerea* are absent from *A. bisporus* strains. *A. bisporus* H97 has a unique gene (colored yellow). Six ORFs are located between the homoedomain genes of *A. bisporus* JB137-S8 but are most likely the result of misassembly.

The locus encoding the homeodomain proteins has previously been located on Chromosome 1 of *A. bisporus* H97 (Morin *et al.* 2012). Our analysis of the mating-type locus of *A. bisporus* ARP23, H97 and JB137-S8 shows they are all very similar to the A mating-type locus of the model species *Coprinopsis cinerea* and are located on scaffold 16, chromosome 1 and scaffold 1 respectively. The locus contains a pair of homeodomain transcription factor genes orthologous to b1-2 and a1-2 from *C. cinerea* (Figure 3). The mitochondrial intermediate peptidase (MIP) gene and a Beta-flanking gene which typically accompany the mating A locus are also found in the genomic vicinity (Figure 3). Interestingly, all three *A. bisporus* strains have an additionally copy of the Beta-flanking gene relative to *C. cinerea*. Levels of synteny with respect to other flanking genes are also very high (Figure 3). The JB137-S8 assembly contains six ORFs between the homeodomain proteins, sequence analysis leads us to believe that this is a misassembly artifact, furthermore all ORFs have homologs in H97 and ARP23 but are located on different scaffolds. Relative to the other mating loci of the other two assemblies, H97 has an additional putative ORF (Figure 3). A homolog for this gene is absent for the assemblies of both ARP23 and JB137-SB, furthermore it does not contain any known Pfam domains and a BLASTP search against GenBank retrieves a single significant hit to another Agaricales species (*Leucoagaricus* sp) therefore it may be dubious.

### Pangenome analysis of Agaricus bisporus

**Figure 4:**
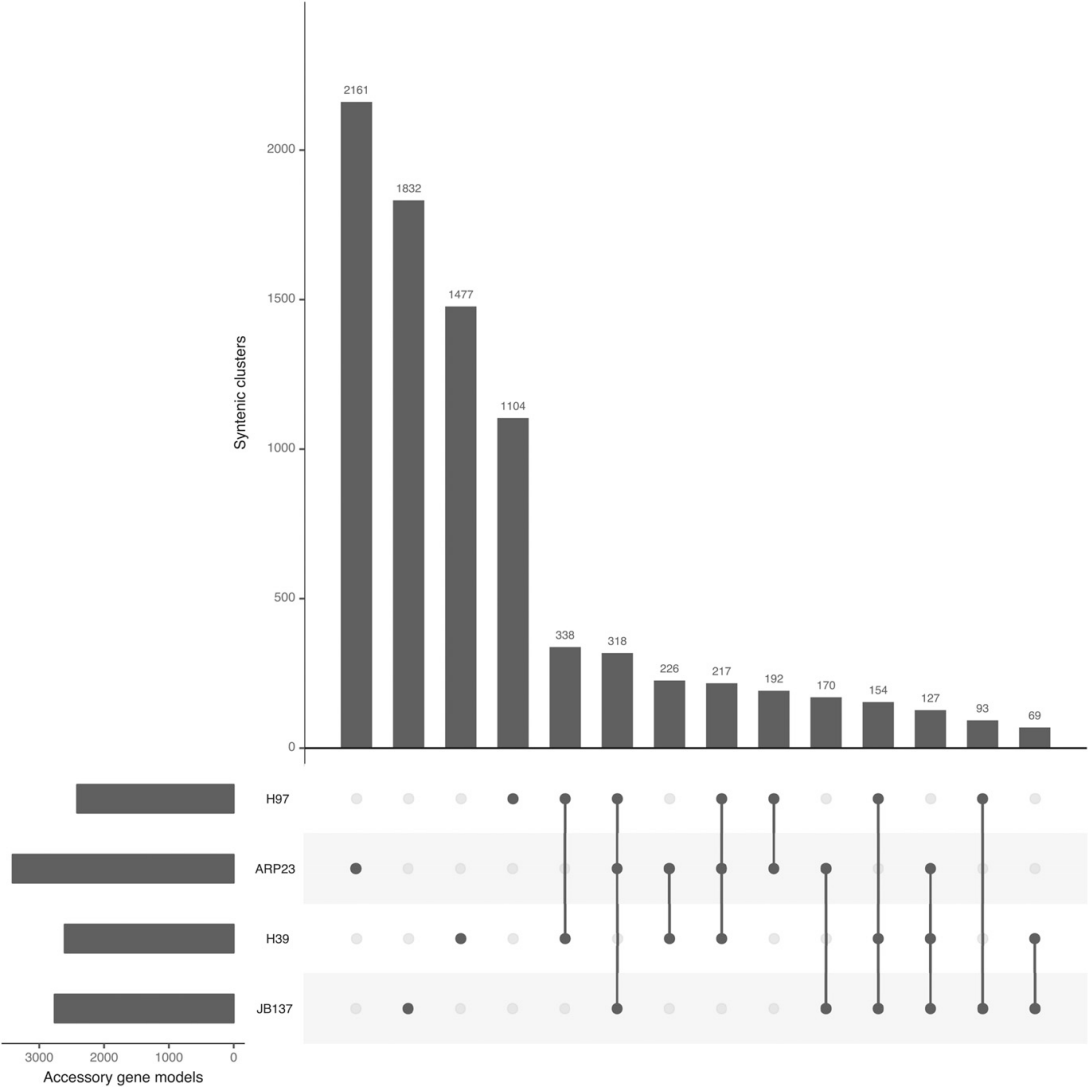
UpSetR plot of the distribution of syntenic orthologous clusters (SOCs) within the accessory genome of *Agaricus bisporus*.

Individual reference genomes do not and cannot contain all genetic information for a species due to genetic and genomic variation between individuals within a species. To account for such variation, it has become increasingly common to refer to species with multiple genomes sequenced in terms of their Pangenome, which is defined as the union of all genes observed across all isolates/strains of a species. A species pangenome for *A. bisporus* was constructed using the synteny-dependent PanOCT method implemented in Pangloss with the default parameters (Fouts *et al.* 2012; McCarthy and Fitzpatrick 2019a, 2019b). PanOCT clusters homologous sequences into syntenic orthologs clusters (SOCs) based on BLAST score ratio (BSR) assessment of sequence similarity and on proportions of relative synteny (conserved gene neighborhood, CGN) between potential orthologs (Rasko *et al.* 2005; Fouts *et al.* 2012). SOCs with syntenic orthologs from all four *A. bisporus* strain genomes in our dataset were classified as “core” SOCs, and clusters missing an ortholog from ≥1 strain genome were classified as “accessory” SOCs. After initial construction with PanOCT, the *A. bisporus* pangenome was refined by merging accessory SOCs based on reciprocal strain best hits between all members of a given pair of accessory SOCs (McCarthy and Fitzpatrick 2019a, 2019b). In total, we identified 7,732 core SOCs and 8,478 accessory SOCs within our *A. bisporus* dataset (16,120 in total) (Figure 4). The proportion of core SOCs relative to the total number of protein coding genes per genome ranged from a low of ∼60% in ARP23 to a high of 71% in H97. This proportion of core to accessory genes is lower than we have previously observed in a number of model fungal species (McCarthy and Fitzpatrick 2019a). Analysis of the distribution of SOCs within the *A. bisporus* accessory genome was performed within Pangloss using UpSetR, which is an R implementation of the UpSet method for visualization of set intersections and occurrences within a dataset using matrix representation. The UpSetR plot in Figure 4 shows that singleton SOCs (*i.e.*, singleton genes) are the most common within the accessory genome, with 2,161 singleton SOCs from ARP23 alone and 6,574 in total (∼78% of all syntenic SOCs in the accessory genome). The distribution of the remaining 1,904 non-singleton SOCs within the *A. bisporus* accessory genome appears to follow evolutionary history (Figure 2), for example H97 and ARP23 share 727 accessory SOCs either exclusively or along with one other strain, while JB137 and ARP23 only share 615 accessory SOCs exclusively or with another strain (Figure 4).

Selection analysis of the core and accessory genomes was performed using the Yang & Nielsen method as implemented in yn00 with the default parameters for yn00. 680 of 7,732 core SOCs (∼9% of core SOCs) and 172 of 1,904 non-singleton accessory SOCs (∼9% of non-singleton accessory SOCs, ∼2% of all accessory SOCs) showed evidence of at least 1 pairwise alignment under positive selection where d_N_/d_S_ ≥ 1 and d_N_/d_S_ ≠ ∞ (Yang and Nielsen 2000).

### Conclusion

In this analysis, we have presented the high quality genome sequence of *A. bisporus* var. *bisporus* ARP23, a commercially relevant genome. In total the genome was found to be 33.49 Mb in length, have high levels of synteny to H39 and contain 13,030 putative protein coding genes. Relative to the other two *A. bisporus* genomes that are currently available, ARP23 is the largest *A. bisporus* strain sequenced to date. Our analyses show that phylogenetically speaking ARP23 is more closely related to H97 than JB137-SB. Furthermore, all three strains have highly conserved mating loci. The lignocellulolytic gene content of all three *A. bisporus* strains is very similar. The pangenome of *A. bisporus* is quite diverse with between 60–70% of genes considered as core SOCs depending on isolate under consideration. The above analyses and genome sequence are the starting point for more detailed molecular analyses into the growth and phenotypical responses of ARP23 when challenged with economically important mycoviruses.
